# Bovine colostrum-derived antibodies against SARS-CoV-2 show great potential to serve as prophylactic agents

**DOI:** 10.1371/journal.pone.0268806

**Published:** 2022-06-10

**Authors:** Kadri Kangro, Mihhail Kurašin, Kiira Gildemann, Eve Sankovski, Eva Žusinaite, Laura Sandra Lello, Raini Pert, Ants Kavak, Väino Poikalainen, Lembit Lepasalu, Marilin Kuusk, Robin Pau, Sander Piiskop, Siimu Rom, Ruth Oltjer, Kairi Tiirik, Karin Kogermann, Mario Plaas, Toomas Tiirats, Birgit Aasmäe, Mihkel Plaas, Karl Mumm, Dagni Krinka, Ene Talpsep, Meelis Kadaja, Joachim M. Gerhold, Anu Planken, Andres Tover, Andres Merits, Andres Männik, Mart Ustav, Mart Ustav

**Affiliations:** 1 Icosagen Cell Factory OÜ, Õssu, Kambja vald, Tartumaa, Estonia; 2 Institute of Technology, University of Tartu, Tartu, Estonia; 3 Department of Clinical Veterinary Medicine, Institute of Veterinary Medicine and Animal Sciences, Estonian University of Life Sciences, Tartu, Estonia; 4 Teadus ja Tegu OÜ, Märja, Tartumaa, Estonia; 5 Chemi-Pharm AS, Tänassilma, Harjumaa, Estonia; 6 Institute of Pharmacy, Faculty of Medicine, University of Tartu, Tartu, Estonia; 7 Institute of Biomedicine and Translational Medicine, Laboratory Animal Centre, University of Tartu, Tartu, Estonia; 8 Ear Clinic of Tartu University Hospital, Tartu, Estonia; 9 Icosagen AS, Õssu, Kambja vald, Tartumaa, Estonia; 10 North-Estonian Medical Centre, Tallinn, Estonia; Qatar University, QATAR

## Abstract

Severe acute respiratory syndrome coronavirus 2 (SARS-CoV-2) continues to impose a serious burden on health systems globally. Despite worldwide vaccination, social distancing and wearing masks, the spread of the virus is ongoing. One of the mechanisms by which neutralizing antibodies (NAbs) block virus entry into cells encompasses interaction inhibition between the cell surface receptor angiotensin-converting enzyme 2 (ACE2) and the spike (S) protein of SARS-CoV-2. SARS-CoV-2-specific NAb development can be induced in the blood of cattle. Pregnant cows produce NAbs upon immunization, and antibodies move into the colostrum immediately before calving. Here, we immunized cows with SARS-CoV-2 S1 receptor binding domain (RBD) protein in proper adjuvant solutions, followed by one boost with SARS-CoV-2 trimeric S protein and purified immunoglobulins from colostrum. We demonstrate that this preparation indeed blocks the interaction between the trimeric S protein and ACE2 in different *in vitro* assays. Moreover, we describe the formulation of purified immunoglobulin preparation into a nasal spray. When administered to human subjects, the formulation persisted on the nasal mucosa for at least 4 hours, as determined by a clinical study. Therefore, we are presenting a solution that shows great potential to serve as a prophylactic agent against SARS-CoV-2 infection as an additional measure to vaccination and wearing masks. Moreover, our technology allows for rapid and versatile adaptation for preparing prophylactic treatments against other diseases using the defined characteristics of antibody movement into the colostrum.

## Introduction

Severe acute respiratory syndrome coronavirus 2 (SARS-CoV-2) emerged in the Chinese province of Hubei in December 2019 and spread worldwide within a few months, leading to the declaration of a pandemic in March 2020. The disease caused by SARS-CoV-2 was named COVID-19 by the World Health Organization [1]. The signs and symptoms of COVID-19 can range from very mild to severe, and they appear 2 to 14 days after exposure. Symptoms include runny nose, sore throat, dry cough, muscle/joint pain, loss of taste/smell, shortness of breath, fever, chills/shaking, diarrhea, nausea/vomiting, fatigue and/or headache [2]. The impact of SARS-CoV-2 on health care systems and the economy worldwide has been devastating [3]. Even with multiple vaccines on the market, the treatment and prevention of SARS-CoV-2 infection must still be developed. Moreover, the virus is continuously adapting, and new viral variants might escape recognition by vaccine-induced immunity [4]. The most notable variants of concern (VoCs) have emerged in the United Kingdom (Alpha, B.1.1.7), South Africa (Beta, B.1.351), and Brazil (Gamma, P.1) and more recently in India (Kappa, B.1.617.1 and Delta, B.1.617.2) [5–7]. Very recently, the Omicron variant (B.1.1.529) has been added to the list [8].

SARS-CoV-2 belongs to the family *Coronaviridae*, which consists of positive-sense single-stranded RNA (+ssRNA) viruses that are members of the subgenus *Sarbecovirus* (*Betacoronavirus* lineage B) [1]. Similar to most other coronaviruses, the SARS-CoV-2 virion contains four structural proteins: S (spike), E (envelope), M (membrane), and N (nucleocapsid). The N protein binds the RNA genome, and the S, E, and M proteins are localized in the viral envelope [9]. The trimeric S protein comprises monomers that consist of S1 and S2 subunits, and it facilitates the entry of the virus into the host cell [10]. More specifically, the receptor binding domain (RBD) in the S1 subunit is responsible for recognition and binding to angiotensin-converting enzyme 2 (ACE2) [11] on host cells, followed by proteolytic activation by host proteases [12]. Thereafter, the S2 subunit mediates the fusion between the virion envelope and the membrane of the host cell [13]. ACE2 is abundantly localized in the epithelia of the lung and small intestine, providing viral entry into human cells [14, 15].

Neutralizing antibodies (NAbs) have been found to block the entry of pathogens into the cell and thus prevent infection [16, 17]. Moreover, SARS-CoV-1 anti-S antibodies have been shown to play a major role in blocking virus entry in a hamster model, while high titers of anti-N antibodies did not provide any protective immunity [18]. Since the initial encounter between the virus and the host is mediated by the RBD region, the majority of the NAbs are directed against RBD [19], although in some cases, the NAbs might target other epitopes on the trimeric S outside the RBD region as well [20]. Intranasal administration of SARS-CoV-2 neutralizing antibodies has demonstrated protection against infection with SARS-CoV-2 variants [21]. Therefore, finding efficient NAbs that could block the entry of SARS-CoV-2 provides a promising approach for developing prophylactic and/or therapeutic means to fight the pandemic. Passive immunization [22] with anti-SARS-CoV-2 NAb could be especially valuable for certain populations that are suffering the most: the elderly, immunocompromised individuals, patients in nursing homes and long-term care facilities, and chronically ill patients.

The passing of protective immunity through colostrum in mammals is a naturally evolved process that provides protection against exogenous pathogens to the newborn [23–25]. Ungulates cannot transfer immunoglobulins across the placenta, and the intestine of a newborn is permeable to proteins for only up to 24 hours after birth [23]. Consequently, the colostrum must contain elevated levels of immunoglobulins that form up to 70–80% of the total protein [26]. Maternal serum immunoglobulins decrease rapidly before parturition and are transported into the colostrum [27], with the main immunoglobulin class being IgG (up to 90%), followed by IgM and IgA [24]. The IgG levels in bovine colostrum have been found to reach up to 20–200 mg/mL [24, 28, 29].

The use of bovine colostrum as a food supplement in humans [29–31] has been demonstrated to elicit beneficial effects against intestinal pathogens [24]. Therefore, the development of hyperimmune bovine colostrum could be an excellent source of specific antiviral antibodies. It was found that the intranasal administration of colostrum antibodies from cows that were vaccinated with influenza vaccine could protect mice from the development of infection when a sublethal dose was administered [32]. Similar transfer of hyperimmune colostrum-derived protection was been demonstrated against *Clostridium difficile* infection [33]. Moreover, intranasal administration of SARS-CoV-2 human NAb was shown to prevent infection in mice [34]. Therefore, the combination of colostrum-derived antibodies from cows immunized with SARS-CoV-2 S protein in a formulation of an intranasal preparation might provide a powerful, affordable and flexible tool to provide protection in the upper respiratory tract, the portal of entry of SARS-CoV-2 infection. Here, we demonstrate that such a colostrum polyclonal antibody preparation can provide an efficient block against SARS-CoV-2 infection, including several known escape variants [35]. We also describe a nasal spray formulation containing the colostrum antibody preparation and its bioavailability in the human nasal cavity.

## Materials and methods

The following SARS-CoV-2 VoCs were used in the course of the study (Table 1). Given is the source of the reference sequence of the Wuhan variant and mutations introduced according to public databases.

**Table 1:** VoCs used in this study.

SARS-CoV-2 variant	Amino acid modification
Wuhan^a^	
Alpha	HV 69–70 del, Y144 del, N501Y, A570D, D614G, P681H, T716I, S982A, D1118H
Beta	L18F, D80A, D215G, LAL 242–244 del, R246I, K417N, E484K, N501Y, D614G, A701V
Gamma	L18F, T20N, P26S, D138Y, R190S, K417T, E484K, N501Y, D614G, H655Y, T1027I, V1176F
Kappa	G142D, E154K, L452R, E484Q, D614G, P681R, Q1071H
Delta	T19R, G142D, E156G, del_F157-R158 L452R, T478K, D614G, P681R, D950N
Omicron	A67V, del69-70, T95I, G142D, del143-145, del211, L212I, ins214EPE, G339D, S371L, S373P, S375F, K417N, N440K, G446S, S477N, T478K, E484A, Q493R, G496S, Q498R, N501Y, Y505H, T547K, D614G, H655Y, N679K, P681H, N764K, D796Y, N856K, Q954H, N969K, L981F

^a^NCBI Reference sequence: NC_045512.2.

### Immunization of pregnant cows

All animal experimental protocols were approved by the Estonian Project Authorization Committee for Animal Experiments on November 5, 2020, and April 16, 2021 (approval numbers 177 and 191, respectively); all experiments were performed in accordance with the European Communities Directive of September 2010 (2010/63/EU); and the study was performed in compliance with the ARRIVE guidelines. Cows (Estonian Holstein breed) were intramuscularly immunized twice with SARS CoV-2 S1 RBD protein (0.1 mg of antigen per injection) followed by 1 boost with SARS-CoV-2 trimeric S protein (0.5 mg per injection) in proper adjuvant solutions. For adjuvant, a mixture of Quil-A (0.5 mg/mL, Invivogen, Toulouse, France) and Imject Alum (20 mg/mL, ThermoFisher Scientific) was used for the immunization procedure, except for 5 cows for which Quil-A (0.5 mg/mL, Invivogen) alone was used. The initial immunization was performed 56–72 days before the expected calving date. The second immunization was administered three weeks after the first injection, and the boost was performed two weeks after the second injection. After calving, the cows continued to receive reimmunizations, equal to the initial immunizations, with proteins at 0.1 mg or 0.15 mg of different VoCs (Table 1.; Alpha, Beta, Gamma or Delta) once every 5–6 weeks.

### Extraction of antibodies from whey

Colostrum was collected twice daily from each cow, defatted, casein depleted, and stored at -20°C until further purification steps. Prior to these steps, different fractions of this stored whey were first analyzed for the presence of NAbs using a competitive enzyme-linked immunosorbent assay (ELISA; Icosagen, K5-002-096), and the fractions containing NAbs were pooled for the following purification steps. To remove casein, the whey was melted at +4°C, the pH was adjusted to 4.2–4.5 using 1 M HCl, and the whey was left at room temperature (RT) for 1 hour with continuous mixing using a magnet stirrer. Thereafter, the pH was adjusted to 3.3 with 1 M HCl, and the whey was left for 1 hour at RT to inactivate possible viral contaminants. The acid pH-treated colostrum whey was neutralized to a pH of 6.7–7.0 using 1.5 M Tris-HCl with a pH of 8.8 and filtered through a 5-μm filter. The filtrated solution was mixed with ammonium sulfate to a final concentration of 2 M and incubated at +4°C overnight. The protein precipitate was separated from the supernatant by centrifugation at 7000x g for 15 min at 4°C. The precipitated proteins were dissolved in 1x DPBS (Dulbecco’s phosphate-buffered saline) solution and concentrated to 100 mg/mL. The concentration was determined by measuring the UV absorbance at a wavelength of 280 nm (A^0.1%^ 280 nm = 1.37). The concentrated immunoglobulin-enriched solution was filtered through a 1.2-μm prefilter and a 0.22-μm filter and dialyzed against 1x DPBS by means of tangential flow filtration (TFF). TFF was performed on Äkta Flux 6 using Sartocon cassettes (50-kDa cutoff). Dialyzed proteins were sterilized by filtration (0.22-μm filter) followed by pasteurization at 57–58°C for 30 min [36].

### SARS-CoV-2 neutralizing antibody ELISA

The wells of Nunc MaxiSorp flat-bottom 96-well plates were coated with purified SARS-CoV-2 trimeric S protein (Table 1) at 2.5 μg/mL in PBS overnight at 4°C and blocked with a PBS solution containing 1% bovine plasma albumin (BPLA) for 1 hour at RT. The samples were diluted in assay buffer (0.5% BPLA in PBS) and added in a 1-in-2 dilution in triplicate to the blocked plate with a starting concentration of 3.2 mg/mL in a final volume of 50 μL and were preincubated for 20 min at RT. On each 96-well plate, assay buffer containing no antibodies was added in triplicate (negative control). Next, an enzyme complex (50 μL) containing biotinylated ACE2-hFc and Pierce^™^ High Sensitivity Streptavidin HRP (horse radish peroxidase) labeled at a concentration of 0.5 μg/mL was added to the preincubated plates and incubated for 30 min at RT. Colorimetric development was performed by using 3,3’,5,5’-tetramethylbenzidine VII substrate. The reaction was stopped using 0.5 M H_2_SO_4_, and the absorbance was measured at 450 nm. The OD values of the measured samples were divided by the mean value of the three repeats of the negative control to obtain relative OD values.

### Pseudovirus neutralization assay

The pseudovirus assay to measure the entry and integration of lentivirus proviral DNA into ACE2-presenting human cells, dependent on the presence of the SARS-CoV-2 S protein, was performed as described previously [37].

Target cells overexpressing full-length human ACE2 protein were established by stable transfection of HEK-293 cells (ATCC) with the plasmid pLV-ACE2 [38]. This step was followed by hygromycin selection.

HIV particles pseudotyped with the SARS-CoV-2 trimeric S protein were generated by cotransfection of wild-type HEK293 cells. Cotransfection was performed using Lipofectamine 3000 reagent (Thermo Fisher Scientific). The plasmid pNL4-3.luc.R-E (firefly luciferase expressing HIV-1 genome with defective envelope protein) and a codon-optimized expression vector of SARS-CoV-2 S protein (lacking the C-terminal 19 amino acids) of the desired virus variant (see Table 1) were co-transfected. Cells were grown in DMEM (plus 10% FBS and 1% penicillin-streptomycin). Seventy-two hours post-transfection, the pseudovirus-containing culture medium was collected and filtered through a 0.45-μm filter. To test the neutralization potency of the colostrum immunoglobulin preparations, ACE2-overexpressing target cells were seeded in 96-well white polystyrene microplates at 4 × 10^4^ cells/well in DMEM (with 10% FBS and 1% penicillin-streptomycin) and were grown at 37°C with 5% CO_2_. Forty-eight hours later, the medium was replaced with pseudovirus-containing medium alone (control) or supplemented with the appropriate colostrum immunoglobulin preparation dilution series (from a concentration of 1 mg/mL to 0.15 μg/mL). One day later, the pseudovirus-containing medium was replaced with fresh medium, and the cells were incubated for two more days before measuring firefly luciferase activity (metric for pseudovirus entry and integration) using the Steady-Glo^®^ Luciferase Assay System and GloMax reader according to the manufacturers’ instructions (Promega). The experiments were performed using three (immunized cow samples) or two (nonimmunized cow samples) biological repeats.

### Rescue of SARS-CoV-2 recombinant viruses (Alpha and Beta strains) from infectious clones

Double knock-in baby hamster kidney (BHK) cells expressing SARS-CoV-2 N protein and human ACE2 receptor were pre-seeded onto T25 flasks to reach sub-confluency (~90–95%). Five micrograms of plasmid DNA encoding SARS-CoV-2 infectious clones (Alpha or Beta strains; see Table 1) were mixed with 500 μL of OPTI-MEM medium, and 5 μL of PLUS reagent were added to the mixtures. For each mixture, 7 μL of Lipofectamine LTX were mixed with 500 μL of OPTI-MEM. Both mixtures (DNA and Lipofectamine LTX) were combined and incubated for 5 min at RT. During incubation, the growth medium on pre-seeded BHK/SARS-CoV-2 N/hACE2 cells was replaced with 4 mL of viral growth medium (VGM, which is DMEM supplemented with 0.2% BSA and penicillin-streptomycin mix). Transfected BHK cells were incubated at 37°C and 5% CO_2_ in a humidified atmosphere for 24 hours. Next, 1 mL of the medium was transferred to Vero E6 cells pre-seeded onto T25 flasks with 4 mL of VGM. Vero E6 cells were incubated at 37°C and 5% CO_2_ in a humidified atmosphere until ~50% of the Vero E6 cells showed cytopathic effects (up to 10 days). Then, the viral stocks were harvested, clarified by centrifugation, and titrated using an immuno-plaque assay.

### SARS-CoV-2-induced cytopathic effect neutralization assay

A 3-fold serial dilution of the colostrum immunoglobulin preparation (concentration from 100 μg/mL to 2 ng/mL) from immunized cows and (from 30 mg/mL to 5 μg/mL) from a nonimmunized cow was made using VGM medium. Medium without the preparation was used as a positive control for infection. The dilutions were performed in a 96-well plate in duplicate. Thereafter, 100 plaque-forming units (pfu) of the Alpha or Beta SARS-CoV-2 strain were added to each well, and the sample was incubated for 1 hour at 37°C. Next, 4x 10^4^ Vero E6 cells were added to each well. The calculated MOI (multiplicity of infection) was 0.0025 pfu/cell. However the MOI would change due to the different colostrum derived immunoglobulin preparations added to the assay. The plates were incubated for 4 days at 37°C in a humid environment before evaluation of the cytopathic effect caused by viral infection. The same number of cells were grown without the virus as a negative control. After incubation, the cytopathic effect was evaluated microscopically. The experiment was performed in triplicate.

### Formulation of bovine immunoglobulin preparation for nasal spray

The bovine colostrum immunoglobulin preparation in a nasal spray formulation contained 0.1% sodium benzoate, 0.07% citric acid, 5% polyethylene glycol 400, 1.5% glycerol and 1% polyvinylpyrrolidone K30 in 1x DPBS. All excipient concentrations were selected based on the formulation studies using various multicomponent formulation compositions. The nasal spray formulations were tested for pH (digital pH meter), viscosity (Brookfield LVDVNX CP Rheometer), particle size, polydispersity index (Malvern Zetasizer), and sterility (Eur.Pharm.10th; 2.6.1). The selection of viscosity enhancers and their concentration was performed based on their viscosity and droplet size measurements. For the bioavailability study, the colostrum immunoglobulin preparation was prepared at two different concentrations of 0.1 mg/mL and 0.2 mg/mL in the nasal spray formulation.

### Bioavailability of colostrum immunoglobulin preparation

To test the nasal biological availability of the bovine colostrum immunoglobulin preparation, a clinical study of 16 healthy volunteers was conducted in accordance with the Declaration of Helsinki. Approval was granted by the Tartu University Ethics Committee on March 17, 2021 (approval number 336/T-1), and the trial was registered at ClinicalTrials.gov (Identifier: NCT04916574). Written informed consent was obtained from each healthy volunteer.

The study group was divided into two subgroups, in which the individuals were intranasally administered (double spraying; ~100 μL per spray) in both nostrils with a nasal spray formulation that contained either 0.1 mg/mL or 0.2 mg/mL of the colostrum immunoglobulin preparation. One hour and four hours after administration, a sample (from either nostril) was obtained using a filter paper piece with a volume capacity of 15 μL, which was kept on the nasal mucosa (medial surface of the inferior turbinate) for 10 min. As a baseline, a sample from each individual was obtained prior to administration. The samples were dissolved in 235 μL of PBS buffer containing 0.05% Tween 20 and protease inhibitor cocktail (Roche) and analyzed using a Cow IgG ELISA kit (Abcam ab190517). The OD450 values of the baseline samples were subtracted from the OD450 values of the samples collected after nasal spray administration, followed by IgG concentration calculations according to the manufacturer’s instructions.

### Statistical analysis

Statistical analyses were performed with GraphPad Prism software, version 9.1.0. The IC_50_ concentrations for ELISA and the pseudovirus assay were determined using 4-parameter nonlinear regression. Statistical comparisons in the induced cytopathic effect neutralization assay were performed using the unpaired t test.

## Results

### Immunoglobulin preparation from colostrum

**Fig 1:**
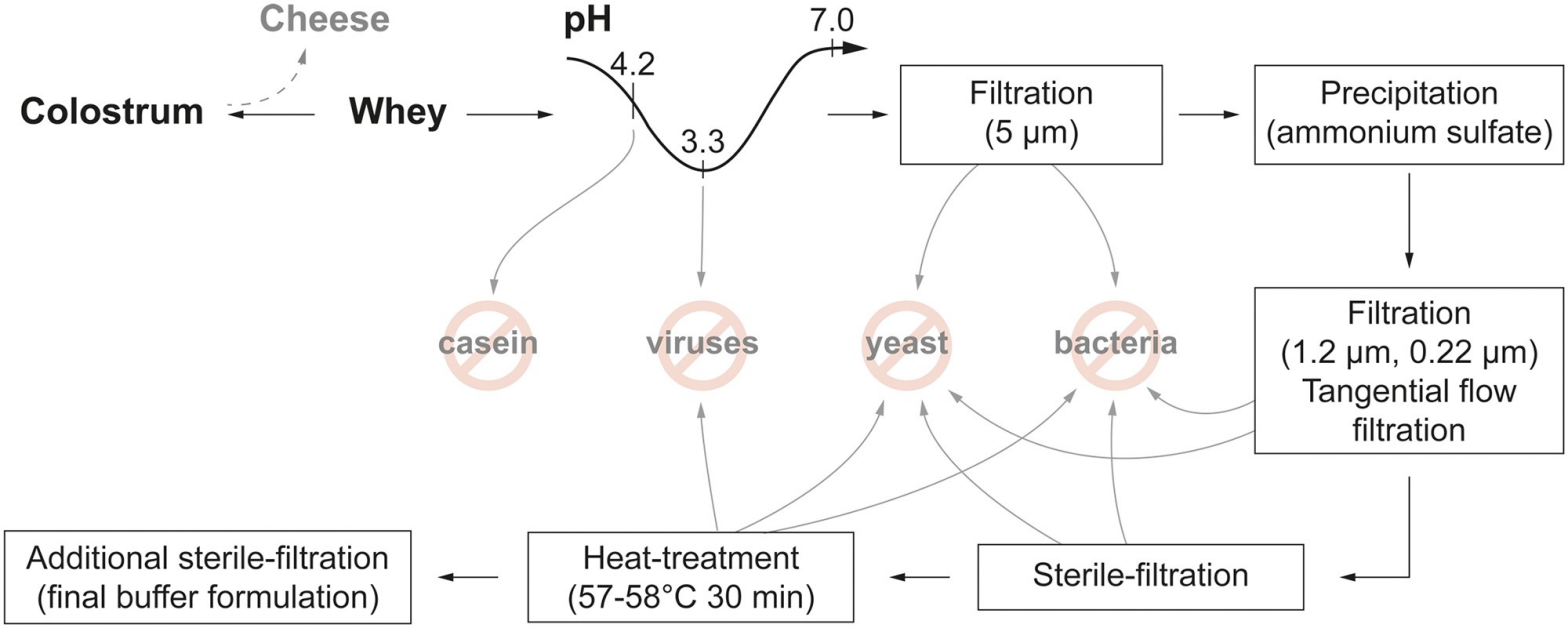
Schematic overview of bovine immunoglobulin preparation from colostrum.

Of 19 immunized cows, only 18 cows reached successful calving and thus provided colostrum. The colostrum was treated with chymosin, and lipids were removed to obtain whey. Since the two separate immunization schemes with different adjuvant mixtures resulted in a rather equal outcome, all of the whey fractions containing NAbs were pooled. The whey was further pH treated, underwent different filtration and fractional precipitation steps and was finally formulated into a buffer solution (Fig 1).

### Colostrum-derived neutralizing anti-S antibodies block trimeric S protein and ACE2 interaction

**Fig 2:**
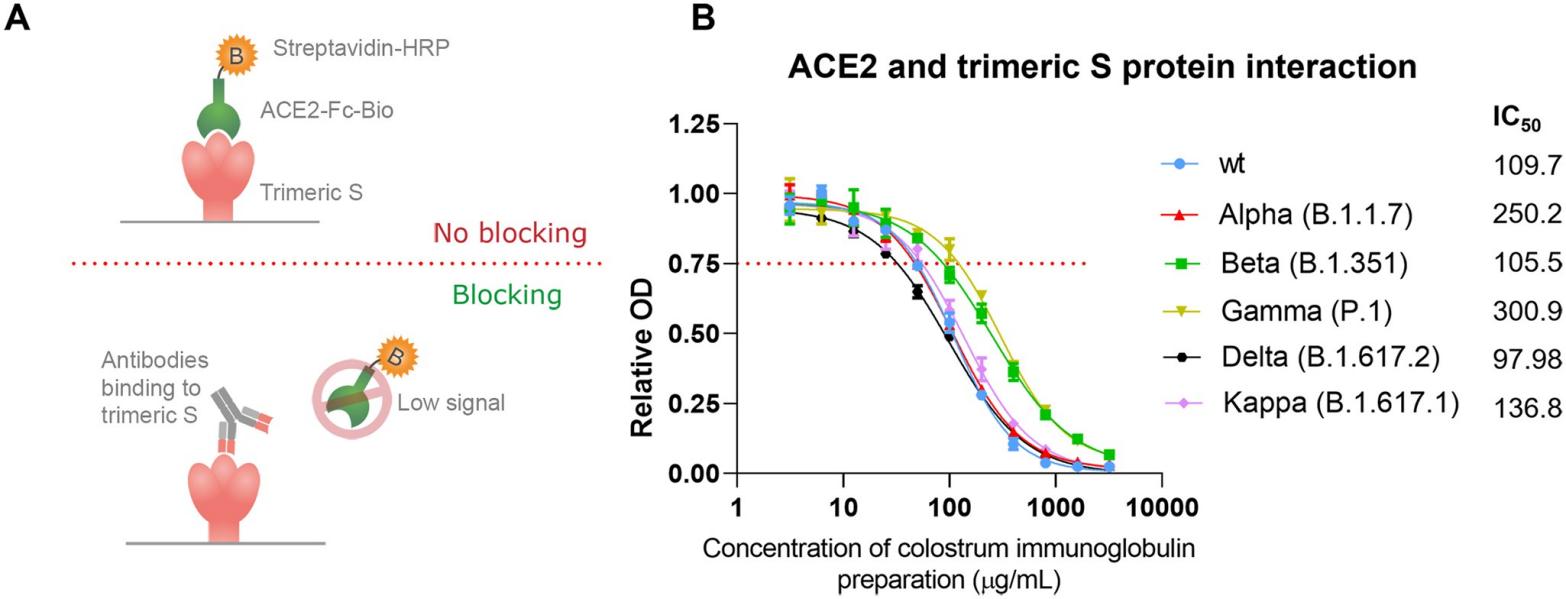
(A) NAb ELISA setup and principle. Colostrum immunoglobulin preparation was added in serial dilution to the trimeric S protein-coated plates and preincubated to provide some time for the antibodies to bind. Next, conjugated ACE2, which competes with the antibodies for the same binding sites, was added. Color development becomes more intense in samples containing fewer neutralizing antibodies, and a sample without any antibodies has the most intense color since maximal binding of conjugated ACE2 could occur. (B) Binding of colostrum immunoglobulin to the different trimeric S protein variants in SARS-CoV-2. Relative optical density (OD) values are represented as the mean ± SD (n = 3). The IC_50_ concentrations for colostrum immunoglobulin preparation were determined using 4-parameter nonlinear regression.

To characterize blocking of the interaction between trimeric S and ACE2 by the colostrum immunoglobulin preparation of different SARS-CoV-2 variants, we determined the interaction blocking efficacy of the antibody preparation using competitive ELISA. The colostrum immunoglobulin preparation was added in a serial dilution to the precoated trimeric S proteins of the wild type (wt), Alpha, Beta, Gamma, Delta or Kappa VoCs, followed by incubation with conjugated ACE2. Since the antibodies and enzyme conjugate are competing for the same binding sites (Fig 2A), the binding rate of the enzyme conjugate revealed whether the colostrum immunoglobulin preparation was able to block the interaction between ACE2 and trimeric S. Relative OD values ≥0.75 were determined to show no ACE2 blocking capacity by the antibodies since the conjugated ACE2 could still bind to the RBD site. Relative OD values <0.75 indicate the ACE2 blocking capacity of the analyzed antibody since the antibodies could bind to the coated S protein and thereby hinder the further binding of ACE2 to the RBD. The half-maximal inhibitory concentration (IC_50_) was calculated for the binding to each trimeric S protein variant. We found that colostrum immunoglobulin preparation blocked ACE2 and wt, Alpha, Beta, Gamma, Delta and Kappa trimeric S interactions with IC_50_ values of 109.7 μg/mL, 250.2 μg/mL, 105.5 μg/mL, 300.9 μg/mL, 97.98 μg/mL and 136.8 μg/mL, respectively (Fig 2B). Thus, the colostrum immunoglobulin preparation should be able to block viral entry into human cells.

### Colostrum immunoglobulins inhibit SARS-CoV-2 S protein-dependent viral entry in a pseudovirus assay

**Fig 3:**
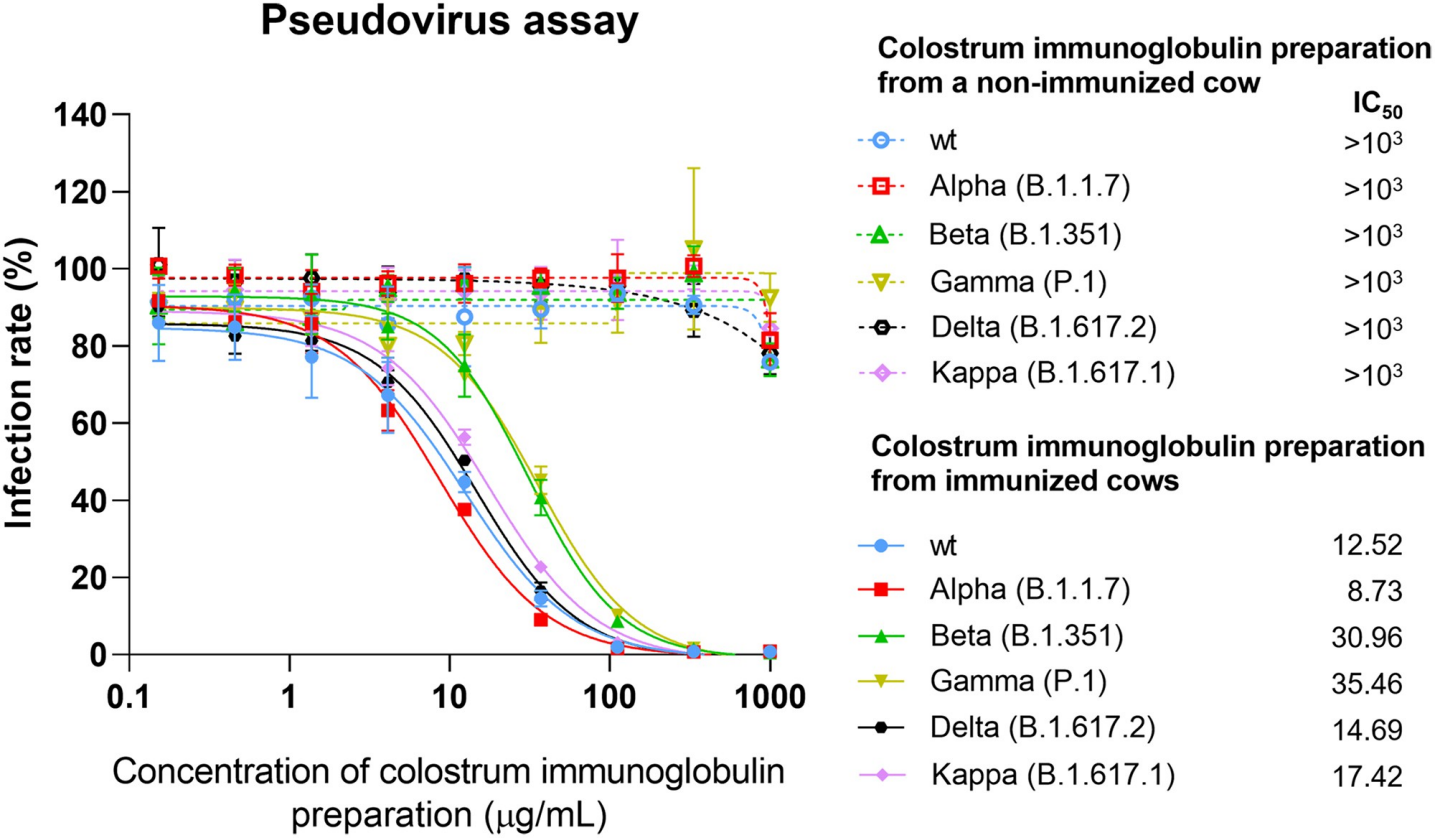
Pseudovirus neutralization assay. Measurement of the inhibition of trimeric S protein-dependent entry of different pseudoviruses pseudotyped with wt, Alpha, Beta, Gamma, Delta or Kappa VoCs of the SARS-CoV-2 S protein into ACE2-expressing HEK293 cells in the presence of serial dilutions of colostrum immunoglobulin preparation. The rate of cell entry was measured via firefly luciferase activity and expressed as the relative infection rate (%) compared to luciferase activity in the untreated control. Data are represented as the mean ± SD (n = 3). The IC_50_ concentrations for colostrum immunoglobulin preparation were determined using 4-parameter nonlinear regression.

To characterize the neutralizing potency of the colostrum immunoglobulin preparation in terms of virus entry, we used a pseudoviral neutralization assay. In this assay, HIV pseudovirion entry into ACE2-expressing target cells is strictly dependent on pseudotyping with the SARS-CoV-2 S protein. The luciferase marker gene incorporated into the HIV genomic sequence becomes active only after successful cell entry and provirus integration. Thus, inhibition of the luciferase signal directly reflects the inhibition of pseudovirus cell entry. Such analyses were performed in the presence of serial dilutions of immunoglobulin preparations. ACE2-expressing HEK293 cells were infected with an HIV-based pseudovirus delivering a luciferase marker gene to the infected cells. These viral particles were pseudotyped with wt, Alpha, Beta, Gamma, Delta or Kappa VoCs of the SARS-CoV-2 S protein. We found that the colostrum immunoglobulins from the nonimmunized cow showed no effective inhibition even at the highest concentration (IC_50_ >10^3^ μg/mL, Fig 3). In contrast, immunoglobulins from immunized cows blocked the entry of pseudoviruses carrying the S protein of wt, Alpha, Beta, Gamma, Delta and Kappa into the cells with IC_50_ values of 12.52 μg/mL, 8.73 μg/mL, 30.96 μg/mL, 35.46 μg/mL, 14.69 μg/mL and 17.42 μg/mL, respectively (Fig 3).

### Colostrum immunoglobulins protect VeroE6 permissive cells from SARS-CoV-2-induced cytopathic effects

**Fig 4:**
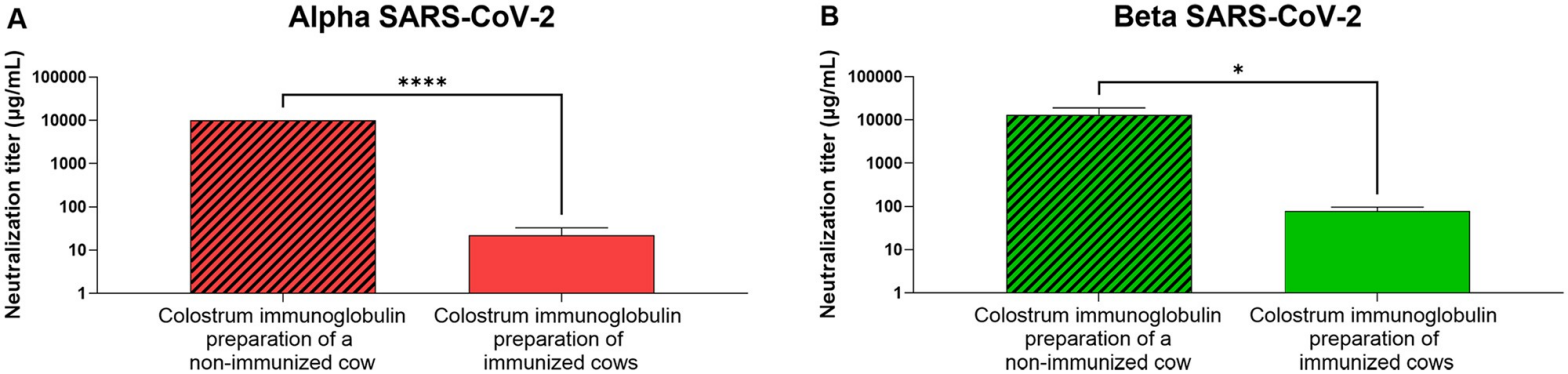
The neutralizing activity of the colostrum immunoglobulin preparation on SARS-CoV-2 infection analyzed by cytopathic effect reduction. Colostrum-derived immunoglobulins from a nonimmunized cow and from SARS-CoV-2 S protein-immunized cows were added to (A) Alpha and (B) Beta SARS-CoV-2 virus isolates at different concentrations and then transferred to Vero E6 cells, which were grown for 4–5 days. The neutralization activity was defined by the endpoint method, i.e., determination of colostrum immunoglobulin preparation concentrations blocking infection of Vero E6 cells. Data are represented as the mean ± SD (n = 3). The unpaired t test was performed; Alpha: ****p<0.0001, Beta: *p = 0.0164.

NAbs can have additional activities (e.g., act via epitopes outside of the receptor binding domain of the S protein) useful for the inhibition of viral infection. Thus, we also tested the inhibitory effect of the colostrum immunoglobulin preparation using authentic Alpha and Beta SARS-CoV-2 strains and Vero E6 cells, which are highly susceptible to SARS-CoV-2 infection, resulting in prominent cytopathic effects (morphology change, detachment from the substrate). An immunoglobulin preparation from a nonimmunized cow failed to protect cells from Alpha and Beta SARS-CoV-2 strains at concentrations lower than 10 mg/mL and 30 mg/mL, respectively (Fig 4). The protective concentration of the colostrum immunoglobulin preparation against the Alpha and Beta SARS-CoV-2 strains from immunized cows was observed on average at 22 μg/mL and 77 μg/mL (Fig 4), respectively, significantly lower than the protective concentration of the control colostrum. Thus, although the pregnant cows were immunized with S protein antigen with the sequence respective to the original reference sequence isolated in Wuhan [39], the resulting colostrum immunoglobulin preparation also had neutralizing activity against later emerging virus variants with several mutations in the S protein, including its receptor binding domain.

### Continuation of immunization of cows after the calving period produces efficient immune responses and antibody titers that promise efficient development of immunoglobulins in the whey of regular milk

**Fig 5:**
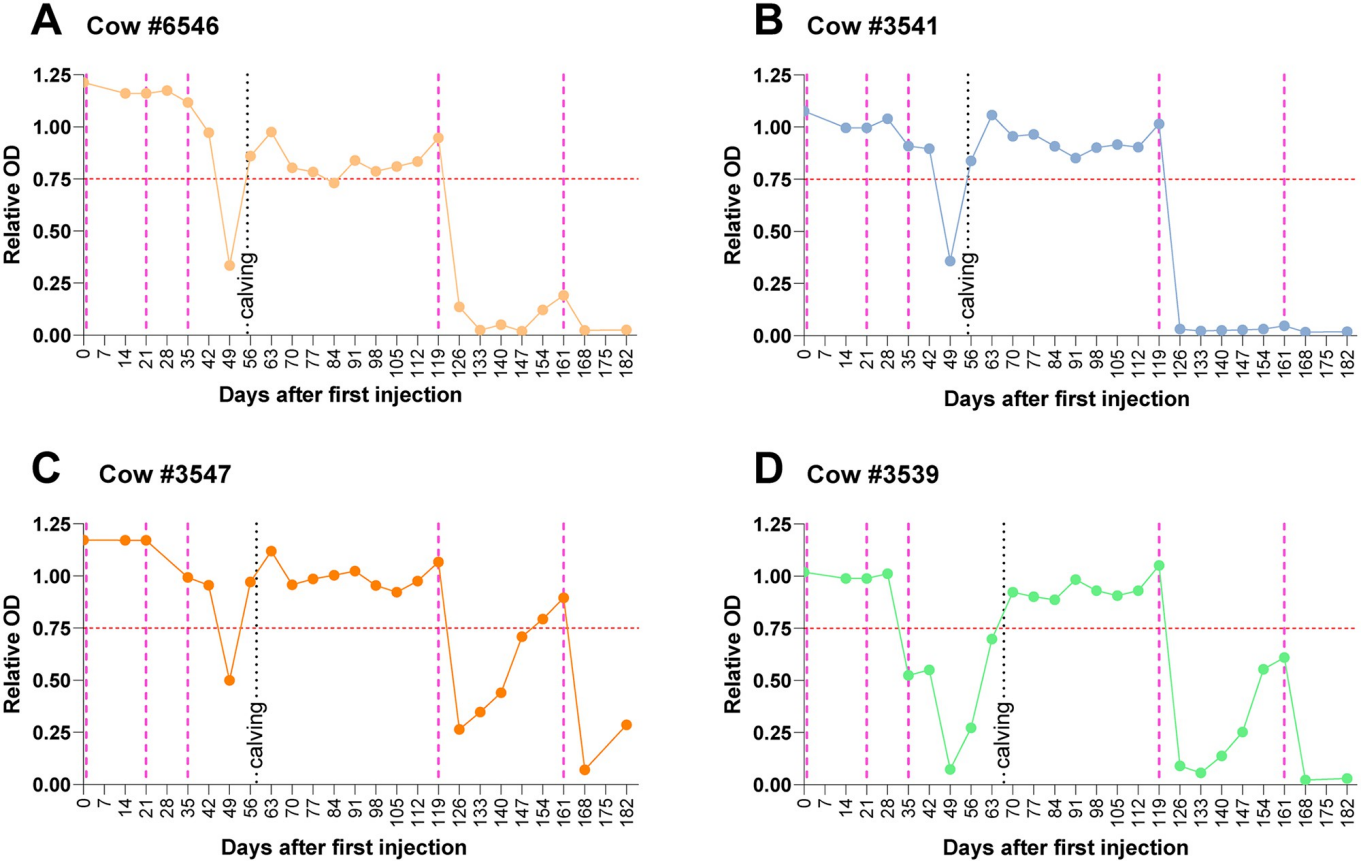
Blood serum neutralizing antibody titers over time after immunization and reimmunization of cows #6546, #3541, #3547 and #3539. The first three pink dotted lines indicate the days of initial immunizations before the first calving. The black dotted lines denote the days of calving, and the following two pink dotted lines indicate reimmunizations. The analyses were conducted using competitive ELISA (see the materials and methods section), and the red dotted line indicates the respective OD 450 of 0.75 (y-axis), above which neutralizing antibodies are not detectable and below which their presence is measurable. The x-axis shows the days after the initial injection for immunization. The connected line shows dots on each day that blood was drawn. This figure shows 4 examples of analyses that were indeed conducted for all cows included in the study.

Employing the ACE2-trimeric S interaction blockage assay, we monitored the efficiency of reimmunzation of cows after calving. Fig 5 depicts the graphs of blood serum analyses of 4 cows that are representative of the entire cohort. Day 0 marks the initial injection in the third trimester of pregnancy, as described before. Follow-up injections were administered 21 and 35 days later, and the gray dotted line depicts the day of parturition. During this period, we were already able to observe that the presence of antibodies blocking ACE2-trimeric S protein interaction in the blood serum, correlating well with the occurrence in the colostrum. Then, 119 days after the initial injection, a booster was administered, upon which a clear response could be observed on Day 126 for all individual cows, albeit with different efficiencies (compare, e.g., #3547 and #3541, for which the difference is clearly observable). The following samples collected at later timepoints showed persistence of the antibodies, whereas titers developed individually over time. Cow #3541, for example, did not show signs of antibody response waning, while cow #3547 barely revealed ACE2-tmerictrimeric S interaction blocking capacity in the sample collected on Day 147. All cows were reinjected on Day 161 after the initial dose. Samples collected on Day 168 again showed a strong immune response, except for the example of cow #3541, the response of which remained unchanged at the highest detectable level throughout the period of observation. Together, we conclude that reimmunization of cows evokes a clear immune response revealing ACE2-TriS interaction blocking antibodies. Reimmunization at an interval of approximately 5–6 weeks produces promising results to predict the steady occurrence of antibodies in milk.

### Diverse boosting during immunization provides colostrum antibodies effective against novel highly mutated virus variants

**Fig 6:**
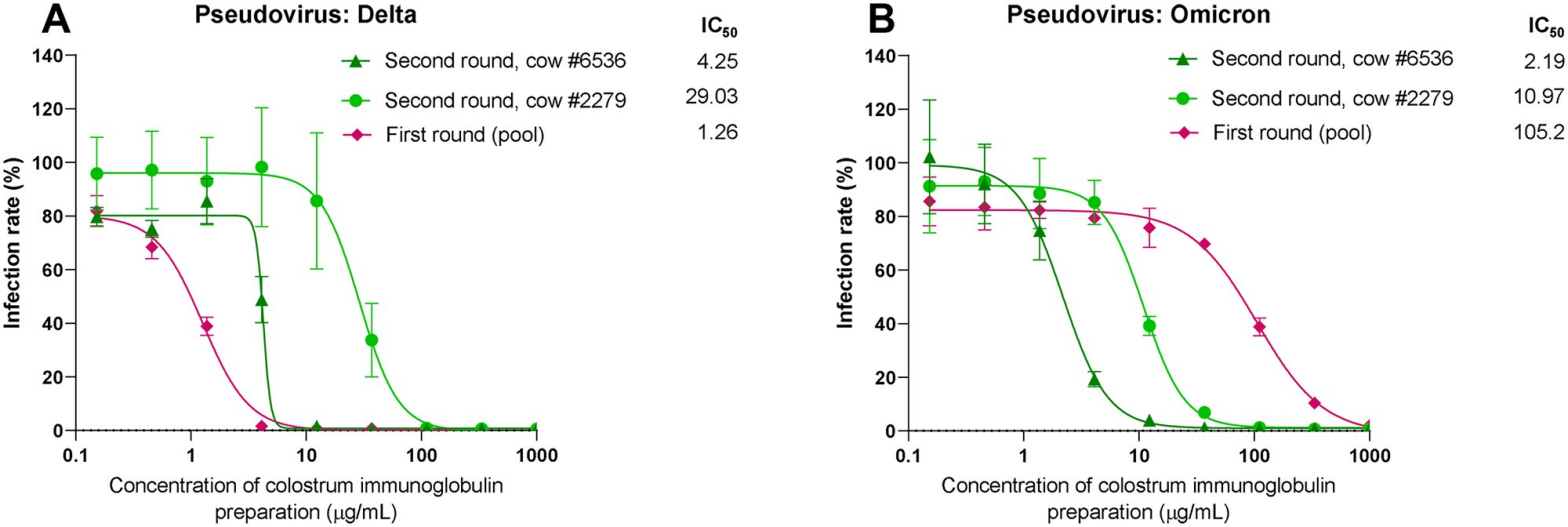
As in Fig 3, the results of the pseudovirus neutralization assay are depicted. Here, pseudoviruses were pseudotyped with either Delta or Omicron VoCs of the SARS-CoV-2 S protein into ACE2-expressing HEK293 cells in the presence of serial dilutions of colostrum immunoglobulin preparations. As described, the rate of cell entry was measured via firefly luciferase activity and expressed as the relative infection rate (%) compared to luciferase activity in the untreated control. Data are represented as the mean ± SD (n = 2–4). The IC_50_ concentrations for colostrum immunoglobulin preparation were determined using 4-parameter nonlinear regression.

After collection of the first round of colostrum immunoglobulins stemming from immunization of the S proteins of the Wuhan isolate, reimmunizations of the animals were continued. The second pregnancy after the very first immunization in the previous year provided the second-round colostrum upon calving. Importantly, as soon as the sequence information about S1 protein of novel variants of concern became available, the variants were used alone or in combination to replace the Wuhan variant in the immunogen for booster dosages. At the end of 2021, when the Omicron variant of the virus arose globally [8], we had colostrum preparations from 2 cows (#6536, #2279) reimmunized 2 to 3 more times between the first and second rounds of colostrum and an additional 3 times before calving of the second round using the S proteins of the Alpha, Beta, Gamma and Delta variants of the virus. The virus neutralization activity of the second-round colostrum was tested using the S proteins of the Delta and Omicron variants in the pseudovirus assay, as described above. As illustrated in Fig 6, the colostrum preparations from those diversely boosted animals but not from the first round of colostrum were highly effective even against the Omicron variant, which was not directly included in immunization. As depicted in Fig 6A, the first immunoglobulin preparation from the first round of immunizations showed an IC_50_ of 26.53 μg/mL on Delta S proteins, while the IC_50_ of the same preparation declined to 105.2 μg/mL on Omicron S proteins (Fig 6B). Conversely, cows #6536 and #2279 revealed equal or better efficiency on Delta S proteins (IC_50_ of 4.25 and 29.03 μg/mL), and these cows stayed comparably well (if not slightly improved) on the Omicron variant (IC_50_ of 2.19 and 10.97 μg/mL, respectively). Omicron has more than 10 amino acid mutations in the RBD region of the S protein compared to the Alpha, Beta, Gamma, Delta and Kappa variants and many S protein mutations outside the RBD. Thus, using the proper immunization scheme, the polyclonal mixture of colostrum immunoglobulins can effectively protect the cells against newly raised variants of concern that are not known at the time of immunization and thus cannot be included in the antigen design immediately. The apparent maturation of polyclonal antibodies due to repeated immunization with proteins from different known VoCs therefore boosts the efficacy and makes the resulting immunoglobulin preparation very efficient.

### The immunoglobulin preparation in a nasal spray formulation persists on the human mucosa for several hours

**Fig 7:**
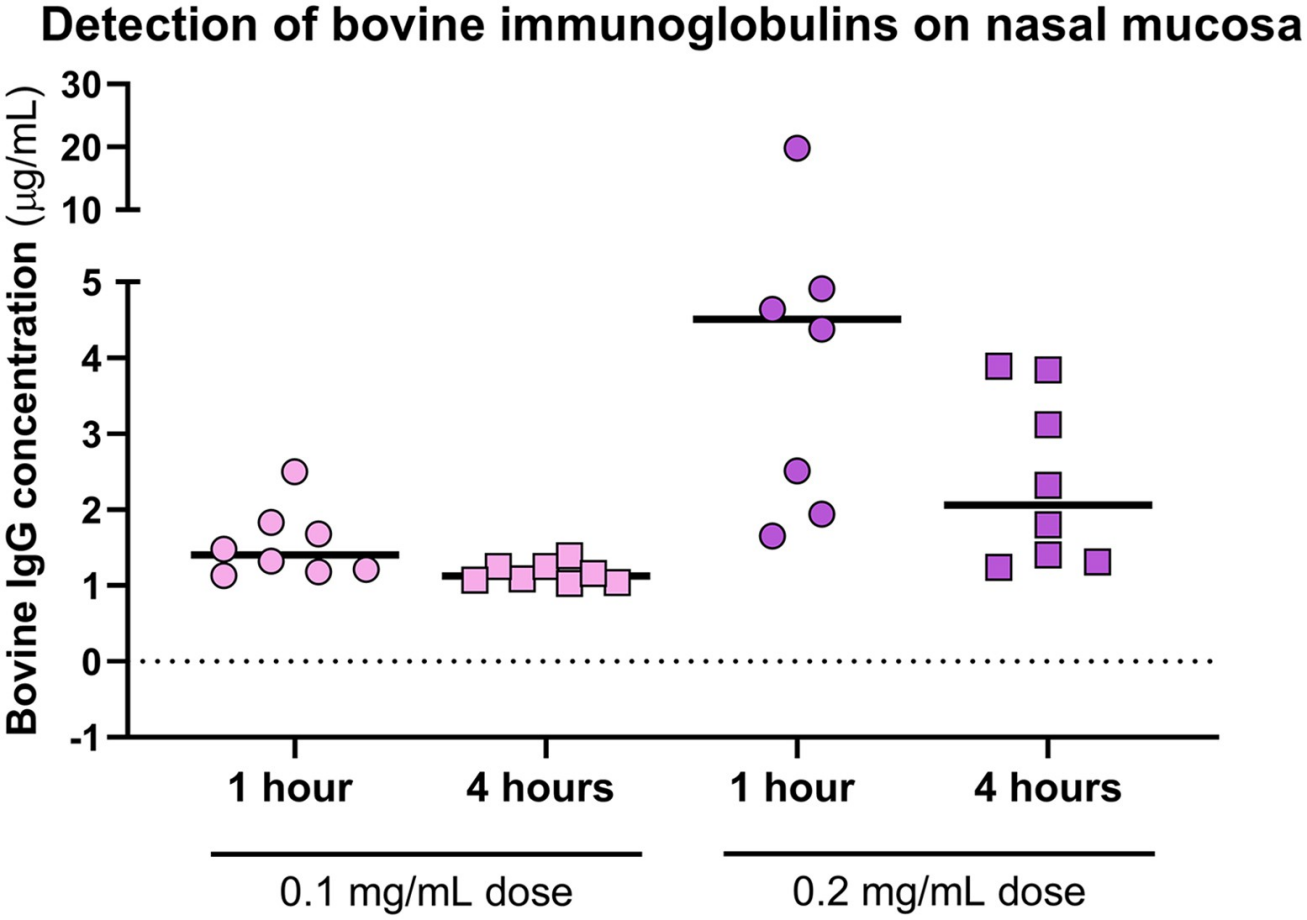
The bioavailability of the final immunoglobulin formulation, which was administered intranasally to healthy volunteers at two different concentrations (0.1 mg/mL and 0.2 mg/mL), and of the residual solution 1 hour and 4 hours postadministration was measured with a cow IgG ELISA kit. The median concentration of bovine IgG is indicated with a black line.

For practical use as a preventive means, the antiviral formulation preferably must be maintained on the site of action for several hours, and the nasal spray dispenser can be used for effective administration of solutions to the upper respiratory tract. To establish the recommended dosage regimen for use and further efficacy studies, we determined how long the immunoglobulin preparation formulation remained on the nasal mucosa. Sixteen healthy volunteers were recruited in a clinical trial. The immunoglobulin preparation as formulated in a nasal spray was administered at two different concentrations by spraying it twice into each nostril. Samples were obtained one hour and four hours after administration to measure the residual bovine immunoglobulin G in the nasal cavities. In the baseline samples collected prior to administration, no bovine IgG was detected. In the subgroup receiving the lower dose (0.1 mg/mL), one hour and four hours after administration, the median bovine IgG concentrations were 1.40 μg/mL and 1.13 μg/mL, respectively (Fig 7). As expected, in the subgroup receiving the higher administered dose, bovine IgG was detected at median concentrations of 4.51 μg/mL and 2.06 μg/mL, respectively (Fig 7). Thus, even at the later time point, the preparation was clearly detectable. No adverse effects were reported by the participants in either of the study groups. Therefore, we concluded that, although bovine antibodies on the nasal mucosa were detectable when applying different doses of colostrum immunoglobulin preparation, a higher concentration is preferred since it allows for a more convenient dosage frequency every 3–4 hours to retain a relevant concentration of antibodies on nasal mucosal surfaces.

## Discussion

Since vaccines are providing protection from the development of lethal COVID-19 [40], the need for additional preventive measures to complement the vaccines to reduce the infection and spread of SARS-CoV-2 is dire. The development and emergency use authorization of monoclonal antibodies for an early infection stage therapeutic and preventive approach has proved to be effective [41]. Issues related to cumbersome administration and immune evasion of the emerging VoCs have reduced the widespread implementation of these therapies. Further studies have indicated that COVID-19 illness has distinctive stages, with indicative clinical findings. Primary infection occurs in the upper respiratory tract epithelium, followed by the second stage of pulmonary disease, with viral multiplication and inflammation in the lungs [42]. By providing an environment in the upper respiratory tract, mucosal surfaces that contain virus neutralizing antibodies can be an attractive strategy for providing protection against early-stage infection. Here, we report that a novel nasal spray containing bovine colostrum-derived antibodies against SARS-CoV-2 shows great potential to serve as a prophylactic agent. This finding adds to the range of measures that are or can be undertaken to fight the spread of SARS-CoV-2.

We demonstrated that immunization of Estonian Holstein breed cows leads to the production of NAbs in their blood serum and that these antibodies efficiently move into the colostrum, from which these antibodies can be easily purified (Fig 1). Apart from this finding, reimmunization at intervals of approximately 5 weeks appeared to produce a continuously efficient antibody titer in the blood serum. Although the respective data are not included here, our preliminary observation indicated that antibodies are consequently detected in the whey of regular milk as well. Using competitive ELISA, which measures the ability of potentially neutralizing antibodies to block the interaction between the RBD on the trimeric S protein and ACE2, we showed not only that this interaction is inhibited by the colostrum-derived immunoglobulin preparation but also that this polyclonal antibody cocktail is also efficient toward different VoCs (Fig 2). This observation was further confirmed in a pseudovirus assay and SARS-CoV-2-induced cytopathic effect neutralization assay, indicating that the polyclonal antibody preparation from colostrum reveals a sufficiently broad specificity against currently widespread SARS-CoV-2 VoCs (Figs 3 and 4). We were able to expand our findings to the recently emerged Omicron variant. The aforementioned reimmunization scheme apparently produced a polyclonal antibody response with mature antibodies of much higher efficiency toward the newly emerged VoC than the initial colostrum-derived immunoglobulin preparation. These results further underscore the great potential of bovine colostrum immunoglobulin preparation as a promising solution to inhibit virus spread, in addition to vaccination. Moreover, the colostrum immunoglobulin preparation remains on the human nasal mucosa for at least 4 hours since the formulation contains viscosity-increasing excipients to prolong the residence and action time on the nasal mucosa and hence assures the successful local delivery of antibody preparation. Preliminary data of anti-S antibody concentrations on the nasal mucosa in patients fully vaccinated with mRNA vaccines showed an average of 2.5 μg/mL [43]. From the colostrum-derived immunoglobulin formulation presented here, if delivered twice per nostril at 0.2 mg/mL, a minimal required concentration is retained at a comparable concentration range on mucosal surfaces after 4 hours, as detected in fully vaccinated individuals [43].

Together, we conclude that the colostrum-derived immunoglobulin preparation provides a very efficient neutralization effect *in vitro*. Our results could be highly significant since it is being reported that polyclonal antibodies from convalescent patients, vaccinated individuals, and different immunized animals might show reduced neutralizing potency to different VoCs of SARS-CoV-2 [17], but a definite neutralization effect has never been reported. Moreover, this technology of colostrum-derived immunoglobulins could be employed for producing (neutralizing) antibodies against various other existing or arising viral diseases and variants thereof.

## Supporting information

S1 Dataset Kangro et al. minimal dataset. (XLSX)
